# Supplementary material for: The Evolution and Characterization of the RNA Interference Pathways in Lophotrochozoa
Source: Genome Biol Evol. 2024 May 7;16(5):evae098. doi: 10.1093/gbe/evae098 (PMC11114477; doi:10.1093/gbe/evae098)
Supplement: evae098_Supplementary_Data [file evae098_supplementary_data.zip › Supplementary_materials.zip!/selected_miRNAs/NoGe_selected_miRNAs/known_miRNA_families_pdf_files/NMRB01000201.1_Notospermus_geniculatus_strain_Ushimado_scaffold201,_whole_genome_shotgun_sequence_7102.pdf]

Provisional ID	:	NMRB01000201.1_Notospermus_geniculatus_strain_Ushimado_scaffold201,_whole_genome_shotgun_sequence_7102
Score total	:	1320.2
Score for star read(s)	:	3.9
Score for read counts	:	1312.4
Score for mfe	:	0.8
Score for randfold	:	0
Score for cons. seed	:	3
Total read count	:	2586
Mature read count	:	2492
Loop read count	:	0
Star read count	:	94

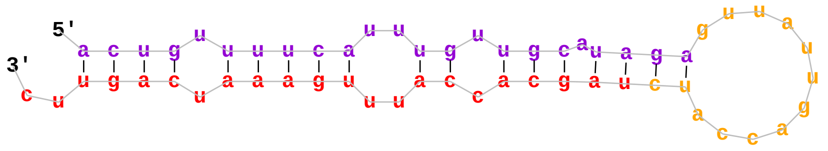

Genome coordinates: NMRB01000201.1 523621-523679

pre-miRNA sequence:
ACUGUUUUCAUUUGUUGCAUAGAACUGUUUUCAUUUGUUGCAUAGAGUUAUUGACCAUCUAGCACCAUUUGAAAUCAGUUC

Mature sequence: UAGCACCAUUUGAAAUCAGUUC

Aligned mature sequence of the *N. geniculatus* pre-miRNA with the top 5 hits from the BLAST search against the pre-miRNA mirGeneDB set:

```
                               ***************** *
nge_Mir-29-P2j                 uagcaccauuugaaaucaguuc-
Tni-Mir-29-P1d1_3p             uagcaccauuugaaaucggugu-
Xbo-Mir-29-P2_3p               uagcaccauuugaaaucagagc-
Xla-Mir-29-P1d4_3p             uagcaccauuugaaaucaguguu
Xtr-Mir-29-P1d_3p              uagcaccauuugaaaucaguguu
```
